# The association between perceived social support and parenting self-efficacy among parents of children aged 0–8 years

**DOI:** 10.1186/s12889-023-16710-8

**Published:** 2023-09-29

**Authors:** Irene N. Fierloos, Dafna A. Windhorst, Yuan Fang, Clemens M. H. Hosman, Harrie Jonkman, Matty R. Crone, Wilma Jansen, Hein Raat

**Affiliations:** 1 Department of Public Health, Erasmus University Medical Center, P.O. Box 2040, Rotterdam, 3000 CA The Netherlands; 2 Department of Cognitive Neuroscience, Donders Institute for Brain, Cognition and Behaviour, Radboud University Medical Center, Nijmegen, The Netherlands; 3 Department of Public Health, TNO Child Health, Leiden, The Netherlands; 4 Department of Health Promotion, Maastricht University, Maastricht, The Netherlands; 5 Department of Clinical Psychology, Radboud University Nijmegen, Nijmegen, The Netherlands; 6 Hosman Prevention and Innovation Consultancy, Berg en Dal, The Netherlands; 7 Verwey-Jonker Institute, Utrecht, The Netherlands; 8 Department of Public Health and Primary Care, Leiden University Medical Center, Leiden, The Netherlands; 9 Department of Youth, City of Rotterdam, Rotterdam, The Netherlands

**Keywords:** Social network, Social relationships, Parenting, Parenting sense of competence, Self-confidence, Self-management

## Abstract

Strengthening social support has been recognized as a potentially effective strategy to enhance parenting self-efficacy, but empirical evidence is limited. This study examined the association between perceived social support and parenting self-efficacy.

Data of 647 parents of children aged 0–8 years, gathered in the CIKEO cohort study in the Netherlands, were analysed. Data were collected between October 2017 and December 2019. Multivariable linear regression models were used to examine the association between social support and parenting self-efficacy. The mean age of the participants was 33.8 years (SD = 4.9); 94.9% mothers. At the start of the study, 15.1% parents perceived low to moderate social support. Parents who experienced lower levels of social support at the start of the study reported lower parenting self-efficacy at follow-up (β: 0.13; 95% CI: 0.05, 0.21), independent of potential socio-demographic confounders. Experiencing an increase in perceived social support during the study period was associated with an increase in parenting self-efficacy (β: 0.15; 95% CI: 0.10, 0.21).

Our findings indicate perceived social support is associated with parenting self-efficacy among parents of children aged 0–8 years. Future longitudinal studies need to confirm our findings and may examine which social support interventions are effective in strengthening parenting self-efficacy.

**Supplementary Information:**

The online version contains supplementary material available at 10.1186/s12889-023-16710-8.

## Introduction

The transition to parenthood is accompanied with many new challenges and can be an overwhelming experience [1]. Many parents have questions or concerns regarding their child’s health, behaviour, development, or their own parenting skills [2]. A study by Glatz and Buchanan [3] indicates that, in the past decades, an increasing amount of parents in high income countries started to feel insecure about their ability to deal with parenting issues. The authors suggest that this may be related to societal changes during this time period, such as changes in expectations of parents, media use for parenting issues and upward social comparison [3]. Also during COVID-19, a decrease in parenting self-efficacy was observed [4]. In several European countries, the demand for specialized youth and family care, including youth mental health care and intensive parenting support, is rising [5–8]. Recently, there has been increasing attention to policies that strengthen parents’ self-efficacy in order to empower them to deal with parenting issues [5, 7, 9]. Parenting self-efficacy can be defined as the extent to which a parent feels confident in dealing with parenting issues [10]. Parenting self-efficacy is important for self-regulation, and has been related to the use of positive parenting practices that promote children’s health and development [11, 12]. Previous studies have shown that parents who perceive higher levels of parenting self-efficacy may be less prone to symptoms of depression, may experience less parenting stress, and may be more persistent in their efforts to deal with difficulties [12]. Children of parents who perceive higher levels of parenting self-efficacy are likely to have more positive beliefs about their own capacities [12].

A theory which has often been applied to parenting self-efficacy is the self-efficacy theory of Bandura [13, 14]. According to Bandura [13], self-efficacy is influenced by four informational sources: 1) past experience, 2) emotional arousal, 3) vicarious experiences (performances of others), and 4) verbal persuasion and feedback [15]. We suggest that three out of four informational sources [2–4] relate to social relationships and social support. Previous studies have shown that social support may reduce emotional arousal [16]. When parents are aware that social support is available, potentially stressful parenting issues may evoke less emotional arousal [17]. Social relationships may also involve an element of social learning: ‘vicarious experiences’ [18]. Seeing how other parents cope successfully with parenting issues may convince parents they too will succeed [19]. Lastly, social relationships may provide verbal persuasion and feedback [20]. Parents who receive positive feedback may feel more confident about parenting [14, 17, 20]. 

According to Cochran and Brassard [21] four types of social support can be distinguished, namely: instrumental, informational, appraisal and emotional support. Instrumental support relates to financial, material and in-kind support; informational support relates to the availability of advice and information; appraisal support relates to the provision of feedback and support with decision-making; emotional support relates to the availability of love, sympathy, esteem, trust, listening and understanding [21–23]. Together, these types form the umbrella concept social support. Based on previous studies, we assume that in particular ‘autonomy-supportive’ social support (i.e. encouraging and accepting the individual), may enhance parents’ psychological well-being and self-efficacy [24, 25]. Negative or controlling social support may have less favourable outcomes [24, 26]. In this study, we focus on examining the role of ‘positive’ autonomy supportive forms of social support in relation to parenting self-efficacy. As a potentially modifiable factor, strengthening positive social support may be a promising strategy to increase parenting self-efficacy [13]. Several previous studies found that higher levels of social support were associated with higher levels of parenting self-efficacy [27–32]. However, other studies found no association [33–35], or found that only specific types of support (i.e. only support provided by a partner, support provided by family, informational support or appraisal support) were associated with parenting self-efficacy [17, 36–40]. These inconsistent findings may be explained by the use of different measures to assess social support and differences in adjustment for potential confounders [17, 31, 32, 29, 34, 36, 38]. Even though several previous studies analysed longitudinal data, they did not examine whether a change in social support was associated with a change in parenting self-efficacy [31, 27–29, 34, 40]. Examining this association over time might provide new insights. 

Also, previous studies paid relatively little attention to different sources of social support and the potential role of anxiety and depression. Experiencing symptoms of anxiety and depression may reduce the likelihood of seeking social support [41] and may be related to relatively more negative perceptions regarding parenting self-efficacy [42, 43]. At the same time, both low levels of perceived social support and low levels of parenting self-efficacy have been associated with increased symptoms of anxiety and depression [32, 43–46]. Due to this interrelatedness between these factors, it may be relevant to take symptoms of anxiety and depression into account when examining the association between social support and parenting self-efficacy.

This study aims to: 1) examine the association between perceived social support at the start of the study and parenting self-efficacy one year later, 2) examine whether a change in perceived social support during the study period is associated with a parenting self-efficacy one year later, 3) explore the potential role of symptoms of anxiety and depression, and 4) explore whether the association between social support and parenting self-efficacy differs between support provided by family, a special person and friends. By studying the change in social support and parenting self-efficacy (aim 2), this study provides empirical insight into the association over time, which is a different perspective compared to previous studies. By paying attention to the potential role of anxiety and depression and specific types of social support (aim 3 and 4), this study may contribute to a better understanding of the association between social support and parenting self-efficacy.

## Methods

### Dataset

This study used data of an observational cohort study embedded in the Consortium Integration Knowledge promotion Effectiveness Of parenting interventions (CIKEO) [47]. The CIKEO study was originally designed to examine associations between (elements of) various types of parenting support and parent and child outcomes, such as preventive parenting programs included in the Dutch ‘Database Effective Youth Interventions’ of the Nederlands Jeugdinstituut [47]. The Medical Ethics Committee of the Erasmus Medical Center, Rotterdam, decided that the rules laid down in the Dutch Medical Research Involving Human Subjects Act (in Dutch: ‘Wet Medisch-wetenschappelijk Onderzoek met mensen’) did not apply to the research proposal (proposal number MEC-2017- 432), that there were no objections to the execution of this study (proposal number MEC-2017- 432), and approved that the results of the study could be submitted to scientific journals (Letter NL/sl/321518; 24/07/2017). The study was conducted in accordance with guidelines and regulations of the Declaration of Helsinki. All participants provided written informed consent. The CIKEO cohort study was registered as NTR7607 in the Netherlands Trial Registry [47].

### Sample/ participants

Participants were recruited between October 2017 and December 2019. Two preventive Youth Health Care providers in the area of Rotterdam and Dordrecht have sent invitation letters to parents/caregivers of children aged 0–8 years in their registries. Questionnaires were returned in a pre-paid envelope or via the internet. Participation was voluntary. All parents who provided informed consent and completed the first questionnaire were enrolled in the study. After 12 months, participants were invited to complete the follow-up questionnaire. 

**Fig. 1:**
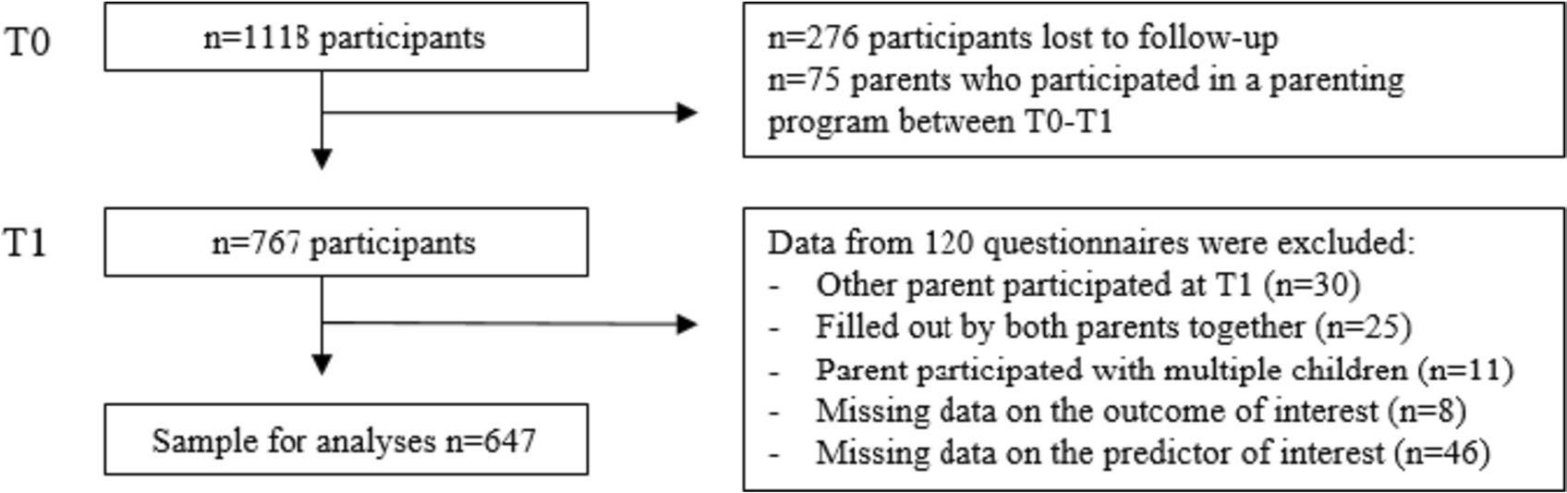
Flowchart of the inclusion process of the CIKEO cohort study and the population for analysis (*n* = 1118)

In total, 1118 parents participated in the first measurement at the start of the study (Fig. 1), we will refer to the first measurement as the ‘baseline’ measurement. In the second measurement, approximately 12 months later, 842 parents participated, we will refer to the second measurement as the ‘follow-up’ measurement. Data from 75 parents who participated in a parenting intervention program [47] between the baseline and follow-up measurement were excluded, because this was assumed to be a potential confounder in the current study. Data from 30 participants were excluded because the follow-up questionnaire was not filled out by the same parent; data from 25 questionnaires completed by two parents together were excluded from the analyses; 11 parents participated in the study with multiple children, questionnaires filled out for their second child were excluded. Participants with missing information on the outcome or predictor (*n* = 54) were excluded from the analyses. Hence, the sample for analyses consisted of 647 participants (Fig. 1).

### Data collection

#### Parenting self-efficacy

Parenting self-efficacy was measured with the self-efficacy subscale of the 17-item Parenting Sense Of Competence scale (PSOC), developed by Gibaud-Wallston and Wandersmann [48]. The PSOC consists of two subscales assessing parents’ self-efficacy and their satisfaction with parenting. In previous studies, Cronbach’s alpha coefficients of the PSOC self-efficacy subscale ranged between .68 and .82 [10, 48–50]. In our sample, the Cronbach’s alpha coefficient for the PSOC self-efficacy subscale was .76, which indicates adequate internal consistency [51]. The 7-item subscale measures parenting self-efficacy by items such as: ‘Being a parent is manageable, and any problems are easily solved’. Each item was answered on a 6-point Likert scale, ranging from 1 = ‘strongly agree’ and 6 = ‘strongly disagree’. One missing item was allowed for the subscale. The weighted sum score for parenting self-efficacy was calculated as described in the guidelines; scores of 7 reported the lowest level of parenting self-efficacy and scores of 42 reported the highest level of self-efficacy [48].

#### Social support 

Perceived social support was measured by the 12-item Multi-dimensional Scale of Perceived Social Support (MSPSS) [52]. Results of previous validation studies indicate that the total score and subscales of the MSPSS have a high internal reliability among diverse groups of participants, with Cronbach’s alpha coefficients for the total score ranging between .84 and .93, and for the subscales between .81 and .98 [52–55]. In our sample, the Cronbach’s alpha coefficient for the total score was .91 and ranged between .88 and .92 for the subscales. The 12-item MSPSS consists of three 4-item subscales assessing perceived social support provided by family members, a special person, and friends, by items such as: ‘I get the emotional help and support I need from my family’; ‘I have a special person who is a real source of comfort to me’; ‘I can count on my friends when things go wrong’. Each item was answered on a 7 point Likert scale, ranging from 1 = ‘very strongly disagree’ to 7 = ‘very strongly agree’. The total score and the scores of the subscales were calculated as described in the guidelines; scores of 1 reported the lowest level of support and scores of 7 reported the highest level of support, no missing values were allowed [53].

#### Covariates

The following socio-demographic characteristics were included as potential confounders: age of the responding parent (in years), gender of the responding parent (female/male), educational level of the responding parent, household income, employment status of the responding parent, immigration background of the responding parent, family composition (one-parent family/two-parent family), number of children in the household (one/two/more than two), age (in years) and gender (girl/boy) of the child for whom the questionnaire was completed. The socio-demographic characteristics were self-reported in the first questionnaire. 

The highest completed educational level of the responding parent was categorized based on the International Standard Classification of Education 2011 [56]. Level 0–2 (no education, primary education, lower secondary education) was categorized as ‘low’; level 3–5 (upper secondary to short-cycle tertiary education) was categorized as ‘middle’; level 6–8 (bachelor to doctoral) was categorized as ‘high’ [56]. Net monthly household income was categorized as low (< €2000), middle (€2000–€3200), or high (> €3200) [57]. Employment status was categorized as ‘working fulltime or part-time’, and ‘no paid job’. When the responding parent or one or both of his/her parents were born outside the Netherlands, this was categorized as an immigration background.

#### Symptoms of anxiety and depression

Symptoms of anxiety and depression were assessed with the anxiety and depression subscales of the Brief Symptom Inventory 18 (BSI-18), a widely used scale in clinical and research settings [58]. The BSI-18 consists of 18-items and three subscales: depression, anxiety, and somatization. One item on thoughts of ending your life of the 6-item depression subscale was removed from the questionnaire, because it was perceived to be too invasive for a postal survey. The items were scored on a 5-point scale of distress ranging from 0 (not at all) to 4 (an awful lot). For the subscales, one missing item was allowed [59, 60]. Weighted sum scores for the anxiety and depression subscale ranged from 0 to 24. Higher scores indicate more symptoms of anxiety or depression. In previous studies, the Cronbach’s alpha for the anxiety subscale ranged between .70 and .84 and for the depression subscale between .70 and .88 [59, 61–63]. In our sample, the Cronbach’s alpha coefficient for the anxiety subscale was .75 and for the adapted depression subscale .80, which indicates adequate internal consistency [51].

### Data analysis

Descriptive statistics were used to characterize the participants. Linear regression models were used to examine the association between social support and parenting self-efficacy. Aim 1 was to examine the association between perceived social support at the start of the study and parenting self-efficacy at follow-up. First, we conducted a simple linear regression model examining the association between social support at baseline and parenting self-efficacy at follow up (Model 2.1). Second, this model was adjusted for potential confounders (Model 2.2). Third, this model was adjusted for potential confounders and baseline levels of parenting self-efficacy, in order to examine whether social support at baseline was associated with a change in parenting self-efficacy during the study period (Model 2.3). Aim 2 was to examine whether a change in perceived social support during the study period is associated with parenting self-efficacy at follow-up. First, we conducted a simple linear regression model examining the association between the change in social support during the study period and parenting self-efficacy at follow up (Model 3.1). Second, this model was adjusted for potential confounders (Model 3.2). Third, this model was adjusted for potential confounders and baseline levels of parenting self-efficacy to examine whether a change in social support during the study period was associated with a change in parenting self-efficacy during the study period (Model 3.3). Aim 3 was to explore the potential role of symptoms of anxiety and depression. First, we conducted a simple linear regression model examining the association between the change in social support during the study period and parenting self-efficacy at follow up, while including symptoms of anxiety and depression in the regression model (Model 4.1). Second, this model was adjusted for potential confounders (Model 4.2). Third, this model was adjusted for potential confounders and baseline levels of parenting self-efficacy, to examine the association between the change in social support during the study period and parenting self-efficacy at follow-up, while including symptoms of anxiety and depression in the model (Model 4.3). Lastly, three linear regression models were used to examine the associations between specific sources of social support (i.e. support provided by family, a special person and friends) and parenting self-efficacy (Model 5.1–5.3). A fourth linear regression model was used to examine the relative contribution of each source of support (Model 5.4). Standardized betas (β) and 95% confidence intervals (CI) were calculated for each factor. 

In addition to the main analyses, we explored whether the association between overall social support and parenting self-efficacy was similar among various groups of parents. Interaction terms (overall social support at baseline*socio-demographic factor) were separately added to the fully adjusted regression model on the association between overall social support at baseline and parenting self-efficacy at follow-up (Table 2; Model 2.2). A Bonferroni correction for multiple testing was applied (*p* = .05/14 = .004). There were no significant interaction effects (Supplementary Table S1). 

Multiple imputation in SPSS was used to deal with missing values of the covariates. Missing values ranged between 0.3% (*n* = 2) for gender of the child and 5.4% (*n* = 35) for income. Five imputed datasets were created for pooled estimates. The regression analyses were repeated in the non-imputed dataset; the results were similar (Supplementary Table S2). The socio-demographic characteristics of participants who were lost to follow-up or excluded from the sample for analysis due to missing data (*n* = 471) were compared to the socio-demographic characteristics of participants included in the sample for analysis (*n* = 647) using chi-squared tests (Supplementary Table S3). Participants lost to follow-up and excluded participants were older (*p* = .002), more often fathers (*p* = .002), more often had a low educational level (*p* = .005), more often had a low income (< .001), less often had a paid job (*p* = .008), more often had an immigration background (*p* < .001), and more often were single parents (*p* < .001). Data were analyzed in Statistical Package for Social Sciences, version 25 for Windows (IBM SPSS Statistics for Windows, IBM Corp). *P*-values below .05 were considered to be statistically significant. 

## Results

### Characteristics of the sample

Table 1 presents the characteristics of the participants. The mean age of the responding parents was 33.8 years (SD = 4.9); 94.9% were women. The mean parenting self-efficacy score was 32.0 (SD = 4.1).At baseline, 84.9% (*n* = 549) perceived high levels of social support (MSPSS ≥ 5.1), and 15.1% (*n* = 98) perceived low to moderate levels of social support (MSPSS < 5.1). Low to moderate levels of social support were more often reported by fathers (*p* < .001), parents with a low educational level (*p* = .038), parents with lower income levels (*p* = .042), and parents without a paid job (*p* = .010). On average, parents perceiving low to moderate levels of social support reported lower levels of parenting self-efficacy (*p* < .001). Correlations between the variables are presented in Supplementary Table S4.

**Table 1:** Socio-demographic characteristics of 647 parents of children aged 0–8 years participating in the CIKEO study; by overall social support at baseline

	**Overall social support at baseline**			
	Total	High *(MSPSS* ≥ *5.1)*	Moderate/ low *(MSPSS* < *5.1)*	*P*-value
	*n* = 647	*n* = 549 (84.9%)	*n* = 98 (15.1%)	
	mean (SD) n (%)	mean (SD) n (%)	mean (SD) n (%)	
*Age of the parent (in years)*	33.8 (SD = 4.9)	33.7 (SD = 4.8)	34.3 (SD = 5.4)	.267
*Gender of the parent*				** < .001**
Female	614 (94.9%)	528 (96.2%)	86 (87.8%)	
Male	33 (5.1%)	21 (3.8%)	12 (12.2%)	
*Educational level* ^a^				**.038**
High	365 (56.4%)	314 (57.2%)	51 (52.0%)	
Middle	245 (37.9%)	209 (38.1%)	36 (36.7%)	
Low	37 (5.7%)	26 (4.7%)	11 (11.2%)	
*Family income*				**.042**
High (> €3200)	415 (67.8%)	362 (69.7%)	53 (57.0%)	
Middle (€2000-€3200)	164 (26.8%)	132 (25.4%)	32 (34.4%)	
Low (< €2000)	33 (5.4%)	25 (4.8%)	8 (8.6%)	
*Employment status of the parent*				**.010**
Part-time	470 (72.9%)	411 (75.0%)	59 (60.8%)	
Fulltime	70 (10.9%)	57 (10.4%)	13 (13.4%)	
No paid job	105 (16.3%)	80 (14.6%)	25 (25.8%)	
*Immigration background of the parent*				.308
No	574 (88.7%)	490 (89.3%)	84 (85.7%)	
Yes	73 (11.3%)	59 (10.7%)	14 (14.3%)	
*Family situation*				.053
Two-parent family	616 (95.5%)	527 (96.2%)	89 (91.8%)	
One-parent family	29 (4.5%)	21 (3.8%)	8 (8.2%)	
*Age of the child (in years)*	3.2 (SD = 1.9)	3.2 (SD = 1.8)	3.3 (SD = 1.9)	.569
*Gender of the child*				.642
Girl	304 (47.1%)	260 (47.4%)	44 (44.9%)	
Boy	342 (52.9%)	288 (52.6%)	54 (55.1%)	
*Number of children in the household*				.745
One child	198 (30.6%)	166 (30.2%)	32 (32.7%)	
Two children	287 (44.4%)	247 (45.0%)	40 (40.8%)	
More than two children	162 (25.0%)	136 (24.8%)	26 (26.5%)	
*Parenting self-efficacy at baseline (higher)*	32.0 (SD = 4.1)	32.3 (SD = 4.0)	30.5 (SD = 4.4)	** < .001**
*Symptoms of anxiety (more)*	1.9 (SD = 2.4)	1.7 (SD = 2.4)	2.6 (SD = 2.6)	**.002**
*Symptoms of depression (more)*	1.9 (SD = 2.7)	1.6 (SD = 2.4)	3.7 (SD = 3.4)	** < .001**

*P*-values < .05 in bold. *P*-values for continuous variables were based on independent t-tests (high/ low), or one-way analysis of variance (stable/ decreasing/ increasing). *P*-values for categorical variables were based on Chi-squared tests. SD = standard deviation. Missing values: income *n* = 35; employment status *n* = 2; family situation *n* = 2; age of the child *n* = 4; gender of the child *n* = 1; symptoms of depression *n* = 4

^a^ Educational level ‘High’: bachelor, master, doctoral or equivalent; ‘Middle’: upper secondary education, post-secondary non-tertiary education, short-cycle tertiary education; ‘Low’: no education, primary education, lower secondary education

### Regression analyses

Linear regression models were used to address the four aims of this study. These aims and corresponding regression models have been described in more detail in the paragraph ‘data analysis’ in the methods section. Below, we report the results of the regression analyses to address aim 1–4.

#### Social support at baseline and parenting self-efficacy at follow-up (aim 1)

Table 2 presents the linear regression models examining the association between overall social support at baseline and parenting self-efficacy at follow-up. Model 2.1 presents the simple regression model. Higher levels of social support at baseline were associated with higher parenting self-efficacy scores at follow-up (β: 0.13; 95% CI: 0.05–0.21). Model 2.2 presents the regression model adjusted for potential confounders. Higher levels of social support at baseline were associated with higher parenting self-efficacy at follow-up (β: 0.13; 95% CI: 0.05–0.21). By additionally adjusting for parenting self-efficacy at baseline, Model 2.3 explored whether social support at baseline is associated with a change in parenting self-efficacy during the study period; this association was not significant (β: -0.02; 95% CI: -0.08, 0.04).

**Table 2:** Results of the linear regression models on the association between overall social support at baseline and parenting self-efficacy at follow-up among parents of children aged 0–8 years participating in the CIKEO study (*n* = 647)

	**Parenting self-efficacy at follow-up** *(Score range* = *7–42)*		
	Model 2.1: Simple regression model	Model 2.2: Adjusted for potential confounders^a^	Model 2.3: Additionally adjusted for self-efficacy at baseline^b^
	β (95% CI)	β (95% CI)	β (95% CI)
*Overall social support at baseline (higher)*	**0.13 (0.05–0.21)**	**0.13 (0.05, 0.21)**	-0.02 (-0.08, 0.04)
Explained variance (based on adjusted R^2^)	1.6%	5.3%	46.9%

Table is based on the imputed dataset. Standardized Betas (β) and 95% confidence interval (95% CI) from linear regression analysis

^a^ Adjusted for the age of the parent, gender of the parent, educational level, family income, work situation, immigration background of the parent, family situation, age of the child, gender of the child, and the number of children in the household

^b^ Adjusted for the age of the parent, gender of the parent, educational level, family income, work situation, immigration background of the parent, family situation, age of the child, gender of the child, the number of children in the household, and parenting self-efficacy at baseline

#### Change in social support and parenting self-efficacy (aim 2)

At baseline, the mean score for parenting self-efficacy was 32.04 (SD = 4.13); at follow-up the mean score for parenting self-efficacy was 31.76 (SD = 4.09). A paired samples t-test showed that the decrease in parenting self-efficacy between the baseline and the follow-up measurement was significant (*p* = .029). The average score for social support was 5.97 (SD = .82) at baseline, and 5.92 (SD = .93) at follow-up. A paired samples t-test showed that this decrease was not significant (*p* = .099). 

Table 3 presents the linear regression models examining the association between the change in overall social support during the study period and parenting self-efficacy at follow-up. Model 3.1 presents the simple regression model. Increasing levels of social support during the study period were associated with higher parenting self-efficacy scores at follow-up (β: 0.17; 95% CI: 0.09, 0.25). Model 3.2 presents the regression model adjusted for potential confounders. Increasing levels of social support during the study period were associated with higher parenting self-efficacy at follow-up (β: 0.17; 95% CI: 0.09, 0.25). By additionally adjusting for parenting self-efficacy at baseline, model 3.3 explored whether a change in social support during the study period is associated with a change in parenting self-efficacy. Increasing levels of social support during the study period were associated with higher levels of parenting self-efficacy at follow-up (β: 0.15; 95% CI: 0.10, 0.21), independent of potential confounders.

**Table 3:** Results of the linear regression models on the association between the change in overall social support between the baseline and follow-up and parenting self-efficacy at follow-up among parents of children aged 0–8 years participating in the CIKEO study (*n* = 647)

	**Parenting self-efficacy at follow-up** *(Score range* = *7–42)*		
	Model 3.1: Simple regression model	Model 3.2: Adjusted for potential confounders^a^	Model 3.3: Additionally adjusted for self-efficacy at baseline^b^
	β (95% CI)	β (95% CI)	β (95% CI)
*Overall social support at baseline (higher)*	**0.18 (0.10, 0.26)**	**0.19 (0.10, 0.27)**	0.03 (-0.03, 0.10)
*Change in overall social support between baseline and follow-up (increasing)*	**0.17 (0.09, 0.25)**	**0.17 (0.09, 0.25)**	**0.15 (0.10, 0.21)**
Explained variance (based on adjusted R^2^)	4.0%	7.9%	49.0%

Table is based on the imputed dataset. Standardized Betas (β) and 95% confidence interval (95% CI) from linear regression analysis

^a^ Adjusted for the age of the parent, gender of the parent, educational level, family income, work situation, immigration background of the parent, family situation, age of the child, gender of the child, and the number of children in the household

^b^ Adjusted for the age of the parent, gender of the parent, educational level, family income, work situation, immigration background of the parent, family situation, age of the child, gender of the child, the number of children in the household, and parenting self-efficacy at baseline

#### The role of symptoms of anxiety and depression (aim 3)

Table 4 presents the regression models that were used to explore the potential role of symptoms of anxiety and depression regarding the association between social support and parenting self-efficacy. The fully adjusted regression model, Model 4.3, indicates that the association between the change in social support during the study period and parenting self-efficacy at follow-up is significant when including symptoms of anxiety and depression at baseline in the regression models (β: 0.15; 95% CI: 0.09, 0.21). In Model 4.3, symptoms of anxiety were negatively associated with parenting self-efficacy (β: -0.12; 95% CI: -0.19, -0.05). In Model 4.2, adjusted for socio-demographic covariates, both symptoms of anxiety (β: -0.15; 95% CI: -0.25, -0.06) and depression (β: -0.12; 95% CI: -0.22, -0.02) were associated with parenting self-efficacy.

**Table 4:** Results of the linear regression models on the association between the change in overall social support between the baseline and follow-up and parenting self-efficacy at follow-up among parents of children aged 0–8 years participating in the CIKEO study (*n* = 647); adjusted for symptoms of anxiety and depression

	**Parenting self-efficacy at follow-up** *(Score range* = *7–42)*		
	Model 4.1: Simple regression model	Model 4.2: Adjusted for potential confounders^a^	Model 4.3: Additionally adjusted for self-efficacy at baseline^b^
	β (95% CI)	β (95% CI)	β (95% CI)
*Overall social support at baseline (higher)*	**0.10 (0.02, 0.18)**	**0.11 (0.03, 0.20)**	0.02 (-0.05, 0.08)
*Change in overall social support between baseline and follow-up (increasing)*	**0.15 (0.07, 0.23)**	**0.16 (0.08, 0.24)**	**0.15 (0.09, 0.21)**
*Symptoms of anxiety at baseline (more)*	**-0.15 (-0.24, -0.05)**	**-0.15 (-0.25, -0.06)**	**-0.12 (-0.19, -0.05)**
*Symptoms of depression at baseline (more)*	**-0.15 (-0.24, -0.05)**	**-0.12 (-0.22, -0.02)**	0.03 (-0.05, 0.10)
Explained variance (based on adjusted R^2^)	10.0%	12.9%	49.9%

Table is based on the imputed dataset. Missing values for symptoms of depression *n* = 4. Standardized Betas (β) and 95% confidence interval (95% CI) from linear regression analysis

^a^ Adjusted for the age of the parent, gender of the parent, educational level, family income, work situation, immigration background of the parent, family situation, age of the child, gender of the child, and the number of children in the household

^b^ Adjusted for the age of the parent, gender of the parent, educational level, family income, work situation, immigration background of the parent, family situation, age of the child, gender of the child, the number of children in the household, and parenting self-efficacy at baseline

#### Specific types of support and parenting self-efficacy (aim 4)

Table 5 presents the fully adjusted regression models examining the association between specific types of social support (support provided by family/a special person/friends) and parenting self-efficacy at follow-up (Model 5.1–5.3). Increasing levels of social support provided by family (β: 0.14; 95% CI: 0.08, 0.20), increasing levels of social support provided by a special person (β: 0.10; 95% CI: 0.03, 0.16), and increasing levels of social support provided by friends (β: 0.14; 95% CI: 0.08, 0.20) during the study period were associated with higher parenting self-efficacy at follow-up, independent of potential confounders and baseline levels of parenting self-efficacy. In an additional fully adjusted regression model (Model 5.4), we examined the relative contribution of each type of support. This model showed that an increase in social support provided by family (β: 0.09; 95% CI: 0.01, 0.16) and an increase in social support provided by friends (β: 0.10; 95% CI: 0.03, 0.16) were associated with higher parenting self-efficacy at follow-up.

**Table 5:** Results of the linear regression models on the longitudinal associations between (the change in) support provided by family, a special person, friends and parenting self-efficacy among parents of children aged 0–8 years participating in the CIKEO study (*n* = 647)

	**Parenting self-efficacy at follow-up** *(Score range* = *7–42)*			
	Model 5.1: Fully adjusted model support by family^a^	Model 5.2: Fully adjusted model support by a special person^a^	Model 5.3: Fully adjusted model support by friends^a^	Model 5.4: Fully adjusted model including all three types of support^a^
	β (95% CI)	β (95% CI)	β (95% CI)	β (95% CI)
*Support provided by family at baseline (higher)*	0.04 (-0.02, 0.10)			0.03 (-0.05, 0.10)
*Change in support provided by family between baseline and follow-up (increasing)*	**0.14 (0.08, 0.20)**			**0.09 (0.01, 0.16)**
*Support provided by a special person at baseline (higher)*		0.02 (-0.04, 0.09)		-0.01 (-0.09, 0.07)
*Change in support provided by a special person between baseline and follow-up (increasing)*		**0.10 (0.03, 0.16)**		0.02 (-0.06, 0.10)
*Support provided by friends at baseline (higher)*			0.04 (-0.02, 0.10)	0.02 (-0.06, 0.10)
*Change in support provided by friends between baseline and follow-up (increasing)*			**0.14 (0.08, 0.20)**	**0.10 (0.03, 0.16)**
Explained variance (based on adjusted R^2^)	48.4%	47.7%	48.6%	48.9%

Table is based on the imputed dataset. Standardized Betas (β) and 95% confidence interval (95% CI) from linear regression analysis. *P*-values < .05 in bold

^a^ Adjusted for the age of the parent, gender of the parent, educational level, family income, work situation, immigration background of the parent, family situation, age of the child, gender of the child, the number of children in the household, and parenting self-efficacy at baseline

## Discussion

This study examined the association between perceived social support and parenting self-efficacy among parents of children aged 0–8 years. First, we examined the association between perceived social support at the start of the study and parenting self-efficacy at follow-up. Parents who experienced lower levels of overall social support at baseline reported lower levels of parenting self-efficacy at follow-up. After adjusting for parenting self-efficacy at baseline, perceived social support at baseline was not associated with parenting self-efficacy at follow-up, which indicates that the level of social support at baseline was not associated with a change in parenting self-efficacy during the study period. Second, we examined whether a change in perceived social support during the study period was associated with parenting self-efficacy at follow-up. Increasing levels of overall social support between the baseline and follow-up measurement were associated with higher parenting self-efficacy at follow-up, also after adjusting for parenting self-efficacy at baseline. Third, we explored the potential role of symptoms of anxiety and depression with regard to the association between social support and parenting self-efficacy. The association between social support and parenting self-efficacy was significant when taking symptoms of anxiety and depression into account. Fourth, we explored whether the association between social support and parenting self-efficacy differed between support provided by family, a special person and friends. We found that changes in all three sources of social support were associated with parenting self-efficacy. Examining the relative contribution of each source of support showed that in particular changes in social support provided by family and friends were relevant for parenting self-efficacy. 

The results of this study are in line with several previous studies which also found a positive association between social support and parenting self-efficacy [27–32]. However, as described in the introduction, the results of previous studies were inconsistent due to the use of different measures to assess social support and differences in adjustment for potential confounders. The results of our study can best be compared to the results of Angley, Divney [27], Rhoad-Drogalis, Dynia [28] and Haslam, Pakenham [40], which also used longitudinal data and adjusted for potential confounders. These studies reported similar associations: higher levels of (positive) social support were associated with higher parenting self-efficacy. Previous longitudinal studies were mainly conducted among parents of babies, while our study was conducted among parents of children aged 0–8 years, this age range was chosen based on the original aim of the CIKEO study [47]. We did not find a significant interaction effect between the age of the child and social support (*p* = .143) (Supplementary Table S1), which indicates that the association between social support and parenting self-efficacy may be similar among parents of older and younger children aged 0–8 years. This may be examined further in future studies.

As mentioned in the introduction, symptoms of anxiety and depression, social support and parenting self-efficacy are interrelated [41–46]. We found significant correlations between symptoms of anxiety and depression, social support and parenting self-efficacy (all *p*-values < 0.001, Supplementary Table S4). The regression models including symptoms of anxiety and depression showed a significant association between social support and parenting self-efficacy, and significant associations between symptoms of anxiety and depression and parenting self-efficacy. We advise to pay attention to the complex interrelatedness between these factors in future studies in order to gain a better understanding of these associations.

### Methodological considerations

Strengths of this study include the relatively large sample size, the use of validated measures to assess social support and parenting self-efficacy, and the possibility to adjust for potential confounders. There are also limitations. First, the representativeness of the sample was limited. A comparison of the participants’ socio-demographic characteristics with national open data [64] showed that parents with a low educational level, parents with lower income levels, parents with a migration background, and parents living without a partner were relatively underrepresented in the sample. The representativeness of the sample was also affected by participants that were lost to follow-up or excluded due to missing data. Although the statistical power to detect associations may have been reduced by this underrepresentation, we have no rationale to expect that the directions of the associations have been affected. Future studies may expand upon our findings by using large and diverse samples of parents and pay special attention to the inclusion of fathers who are often underrepresented in studies about parenting.

Second, our study design did not allow to examine causality between social support and parenting self-efficacy. We recommend to examine the direction(s) of the association in future longitudinal studies. 

### Recommendations for policy and practice

Our findings indicate that social support may be relevant for parenting self-efficacy. Including social support in parenting interventions is recommended as this may improve parenting self-efficacy and other treatment outcomes [65], and may foster a parent’s ability to self-regulate [11, 66]. Results of a recent meta-analysis indicate that universal parent educational interventions are associated with improvements in parenting self-efficacy, regardless of the duration, although longer programs (ten weeks or more) showed greater improvements in parenting self-efficacy [67]. Many existing parenting intervention programs include elements of social support, such as verbal encouragement and praise, group discussions about parenting issues, interpersonal conflict solving, and communication training [67]. There are multiple ways to strengthen social support in parents [68–73]. For example, professionals may play a role in mobilizing support provided by family, a special person, friends and existing social contacts and train parents’ skills to use available social support [68–73]. In addition, professionals may facilitate contact between parents, for example by organizing dialogues about parenting, which may stimulate parents to exchange support and advice [9, 69, 72].

## Conclusion

Perceived social support is associated with parenting self-efficacy among parents of children aged 0–8 years, participating in the CIKEO cohort study. Lower levels of social support at baseline were associated with lower levels of parenting self-efficacy at follow-up. Increasing levels of perceived social support during the study period were associated with higher levels of parenting self-efficacy at follow-up. The association between social support and parenting self-efficacy was significant when taking symptoms of anxiety and depression into account and was similar for social support provided by family, a special person and friends. Future longitudinal studies need to examine the direction(s) of this association among diverse groups of parents. There is a need to examine which types of social support are most effective to use in intervention strategies aiming to strengthen parenting self-efficacy. In the meantime, health and social care professionals are advised to consider using social support as a strategy to strengthen parenting self-efficacy in order to promote self-regulation and related health and wellbeing of parents and children.

### Supplementary Information

**Additional file 1: Table S1.** Results of the analyses of interaction effects between overall social support at baseline and socio-demographic characteristics among participants of the CIKEO study (*n*=647). **Table S2. **Results of the linear regression models on the association between the change in overall social support between the baseline and follow-up and parenting self-efficacy at follow-up among parents of children aged 0-8 years participating in the CIKEO study (*n*=647); non-imputed dataset. **Table S3. **Non-response analysis among the total group of participants of the CIKEO study (*n*=1118). **Table S4.** Pearson correlations between parenting self-efficacy, social support and the covariates included in this study among 647 participants of the CIKEO study.

## Data Availability

The datasets generated and/or analysed during the current study are not publicly available due to privacy or ethical restrictions but are available from the corresponding author on reasonable request.
