# Supplementary material for: The association between perceived social support and parenting self-efficacy among parents of children aged 0–8 years
Source: BMC Public Health. 2023 Sep 29;23:1888. doi: 10.1186/s12889-023-16710-8 (PMC10541688; doi:10.1186/s12889-023-16710-8)
Supplement: Supplementary file 1 — Additional file 1: Table S1. Results of the analyses of interaction effects between overall social support at baseline and socio-demographic characteristics among participants of the CIKEO study (n=647). Table S2. Results of the linear regression models on the association between the change in overall social support between the baseline and follow-up and parenting self-efficacy at follow-up among parents of children aged 0-8 years participating in the CIKEO study (n=647); non-imputed dataset. Table S3. Non-response analysis among the total group of participants of the CIKEO study (n=1118). Table S4. Pearson correlations between parenting self-efficacy, social support and the covariates included in this study among 647 participants of the CIKEO study. [file 12889_2023_16710_MOESM1_ESM.docx]

# Supplementary files social support and parenting self-efficacy

**Table S1.** Results of the analyses of interaction effects between overall social support at baseline and socio-demographic characteristics among participants of the CIKEO study (n=647).

| **Interaction term** | **P-value of the Beta in the multivariable linear regression model on the association between overall social support and parenting self-efficacy adjusted for potential confounders** |
| --- | --- |
| Social support at baseline*age of the parent | .722 |
| Social support at baseline*gender of the parent | .545 |
| Social support at baseline*low educational level | .796 |
| Social support at baseline*middle educational level | .576 |
| Social support at baseline*low income | .587 |
| Social support at baseline*middle income | .806 |
| Social support at baseline*fulltime job | .961 |
| Social support at baseline*no paid job | .863 |
| Social support at baseline*immigration background | .398 |
| Social support at baseline*one-parent family | .056 |
| Social support at baseline*age of the child | .143 |
| Social support at baseline*gender of the child | .875 |
| Social support at baseline*two children | .009 |
| Social support at baseline*more than two children | .499 |

Table is based on the imputed dataset. P-values were derived by separately adding the interaction terms to the fully adjusted multivariable linear regression model on the association between overall social support at baseline and parenting self-efficacy at follow-up (Table 2; model 2.2). Significant p-values <.004 in bold (Bonferroni correction for multiple testing).

**Table S2.** Results of the linear regression models on the association between the change in overall social support between the baseline and follow-up and parenting self-efficacy at follow-up among parents of children aged 0-8 years participating in the CIKEO study (n=647); non-imputed dataset.

|  | **Parenting self-efficacy at follow-up** *(Score range= 7-42)* | | |
| --- | --- | --- | --- |
|  | Model 3.1: Simple regression model | Model 3.2:  Adjusted for potential confounders^1^ | Model 3.3:  Additionally adjusted for self-efficacy at baseline^2^ |
|  | β (95% CI) | β (95% CI) | β (95% CI) |
| *Overall social support at baseline (higher)* | **0.18 (0.10, 0.26)** | **0.16 (0.08, 0.24)** | 0.01 (-0.06, 0.07) |
| *Change in overall social support between baseline and follow-up (increasing)* | **0.16 (0.09, 0.25)** | **0.16 (0.08, 0.24)** | **0.14 (0.08, 0,20)** |
| Explained variance (based on adjusted R^2^) | 4.0% | 8.3% | 49.2% |

Table is based on the non-imputed dataset. Standardized Betas (β) and 95% confidence interval (95% CI) from linear regression analysis. Missing values covariates: age parent n=38; gender parent n=34; educational level n=39; income n=71; employment status n=40; immigration background n=38; family situation n=6; age of the child n=12; gender of the child n=8.

^1^ Adjusted for the age of the parent, gender of the parent, educational level, family income, work situation, immigration background of the parent, family situation, age of the child, gender of the child, and the number of children in the household.
^2^ Adjusted for the age of the parent, gender of the parent, educational level, family income, work situation, immigration background of the parent, family situation, age of the child, gender of the child, the number of children in the household, and parenting self-efficacy at baseline.

**Table S3.** Non-response analysis among the total group of participants of the CIKEO study (n=1118).

|  | Total | Included in the sample for analysis | Excluded from the sample for analysis |  |
| --- | --- | --- | --- | --- |
|  | n=1118 | n=647 (57.9%) | n=471 (42.1%) |  |
|  | mean (SD) n (%) | mean (SD) n (%) | mean (SD) n (%) | P-value |
| *Age of the parent (in years)* | 34.2 (SD=5.2) | 33.8 (SD=4.9) | 34.8 (SD=5.5) | **.002** |
| *Gender of the parent*  Female   Male | 1005 (92.5%)  77 (7.1%) | 614 (94.9%)  33 (5.1%) | 391 (89.9%)  44 (10.1%) | **.002** |
| *Educational level ^1^*  High   Middle   Low | 590 (54.7%)  404 (37.4%)  85 (7.9%) | 365 (56.4%)  245 (37.9%)  37 (5.7%) | 225 (52.1%)  159 (36.8%)  48 (11.1%) | **.005** |
| *Family income*  High (>€3200)  Middle (€2000-€3200)  Low (<€2000) | 686 (65.5%) 276 (26.4%) 85 (8.1%) | 415 (67.8%) 164 (26.8%) 33 (5.4%) | 271 (62.3%)  112 (25.7%)  52 (12.0%) | **<.001** |
| *Employment status of the parent*  Part-time  Fulltime  No paid job | 748 (69.4%)  126 (11.7%)  204 (18.9%) | 470 (72.9%) 70 (10.9%) 105 (16.3%) | 278 (64.2%) 56 (12.9%)  99 (22.9%) | **.008** |
| *Immigration background of the parent*  No  Yes | 921 (85.3%)  159 (14.7%) | 574 (88.7%)  73 (11.3%) | 347 (80.1%)  86 (19.9%) | **<.001** |
| *Family situation*  Two-parent family  One-parent family | 1037 (93.3%) 75 (6.7%) | 616 (95.5%)  29 (4.5%) | 421 (90.1%)  46 (9.9%) | **<.001** |
| *Age of the child (in years)* | 3.2 (SD=2.0) | 3.2 (SD=1.9) | 3.3 (SD=2.1) | .172 |
| *Gender of the child*  Girl  Boy | 538 (48.5%) 572 (51.5%) | 304 (47.1%)  342 (52.9%) | 234 (50.4%)  230 (49.6%) | .268 |
| *Number of children  in the household*  One child  Two children  More than two children | 351 (31.4%)  495 (44.3%)  272 (24.3%) | 198 (30.6%)  287 (44.4%) 162 (25.0%) | 153 (32.5%)  208 (44.2%)  110 (23.4%) | .731 |

P-values <.05 in bold. P-values for continuous variables were calculated with P-values are based on a one-way analysis of variance and P-values for categorical variables were calculated with Chi-squared tests. SD=standard deviation. Missing values: age parent n=38; gender parent n=34; educational level n=39; income n=71; employment status n=40; immigration background n=38; family situation n=6; age of the child n=12; gender of the child n=8.
^1^ Educational level ‘High’: bachelor, master, doctoral or equivalent; ‘Middle’: upper secondary education, post-secondary non-tertiary education, short-cycle tertiary education; ‘Low’: no education, primary education, lower secondary education

**Table S4**. Pearson correlations between parenting self-efficacy, social support and the covariates included in this study among 647 participants of the CIKEO study.

| *Variable* | *Parenting self-efficacy at follow-up*  Pearson correlation r | *Overall social support at baseline (MSPSS)*  Pearson correlation r |
| --- | --- | --- |
| Parenting self-efficacy at follow-up | 1 | .131^***^ |
| Gender of the parent | -.031 | -.174*** |
| Age of the parent | .055 | -.053 |
| Low educational level | -.025 | -.113** |
| Middle educational level | -.069 | .024 |
| Fulltime job | .055 | -.066 |
| No paid job | -.040 | -.116** |
| Low income | -.011 | -.056 |
| Middle income | -.201** | -.083* |
| Single parent | .065 | -.066 |
| Migration background of the parent | .045 | -.066 |
| Child age | -.010 | -.033 |
| Gender of the child | .026 | .007 |
| Two children | -.030 | .053 |
| More than two children | -.042 | -.009 |
| Symptoms of anxiety at baseline | -.247*** | -.166*** |
| Symptoms of depression at baseline | -.266*** | -.319*** |

*p <.05; ** p <.01; *** p <.001.
